# Responding to Ebola through Visual Poetry

**DOI:** 10.3201/eid2202.AC2202

**Published:** 2016-02

**Authors:** Byron Breedlove

**Affiliations:** Centers for Disease Control and Prevention, Atlanta, Georgia, USA

**Keywords:** art science connection, emerging infectious diseases, art and medicine, about the cover, Responding to Ebola through Visual Poetry, Edward Epp, en plein air, From the Randal Street Apartment, to the South East, Monrovia, Ebola, viruses, visual poetry, West Africa, Liberia

**Figure:**
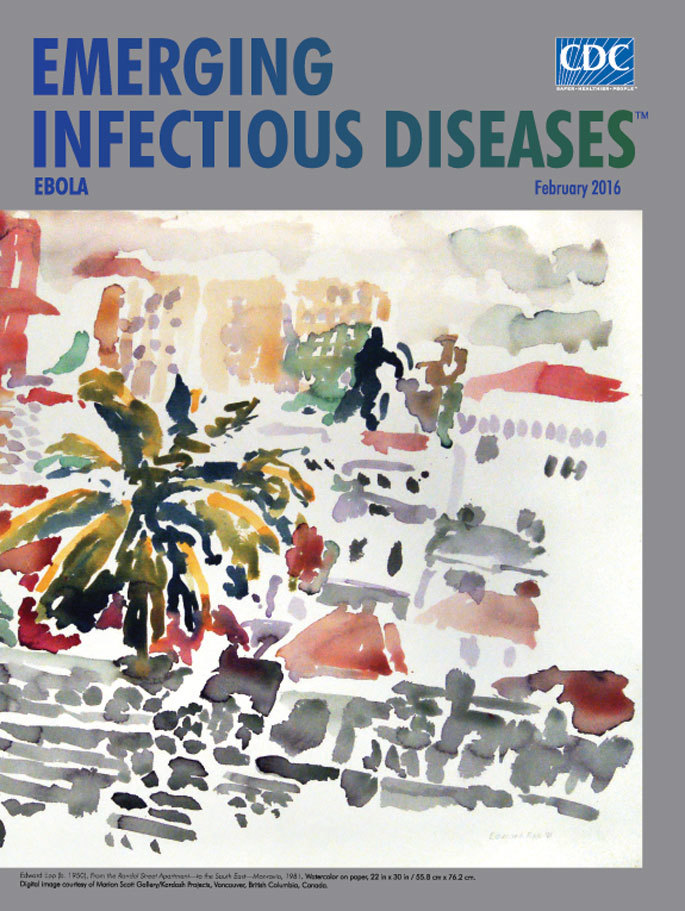
**Edward Epp (b. 1950), *From the Randal Street Apartment—to the South East—Monrovia,* 1981. Watercolor on paper, 22 in x 30 in/55.8 cm x 76.2 cm.** Digital image courtesy of Marion Scott Gallery/Kardosh Projects, Vancouver, British Columbia, Canada

In 1981, when Canadian artist Edward Epp and his wife Leanne Boschman moved to Liberia to work as teachers, they initially lived in an apartment on Randal Street in downtown Monrovia. From there, Epp noted, “the sights and sounds from the community were unlike anything that I had experienced before in North American or European cities: the architecture, the flora, the street noises and outdoor activities, such as football games in a field visible beyond the cement block businesses and private apartments, were all new to us.”^1^

Epp is best known for his light-filled landscapes, and his works have been shown in exhibitions across Canada and in the United States and Botswana. Before traveling to Liberia, he experimented with watercolors to paint scenes from the dense forests of British Columbia, “making abstract patterns and rhythms, very much like jazz-improvisation, suspending judgment, and being open to new environments.”^ 1 ^An advocate of painting en plein air, which stresses attention to color, light, and movement, Epp states in the introduction to his Marion Scott Gallery exhibit that “In Liberia, I was experiencing a new world of light and colour, and the only way for me to make these experiences meaningful was to begin painting what I was seeing and perceiving.”

Explaining that “Painting was and still is a way for me to ground myself in new spaces,”^ 1 ^Epp completed this month’s cover image, *From the Randal Street Apartment—to the South East—Monrovia,* soon after arriving in Liberia. It offers an impressionistic snapshot of the shimmering shapes and colors that spilled out from his vantage point in the brilliant tropical heat and light. The artist’s brush strokes telegraph a sense of bustle, energy, and heat from the city. Edifices, rooftops, and streets are sketched mostly in reds and grays, whereas white spaces define and suggest structure and direction. The green and gold fronds splay out like fireworks from the trunk of a large palm tree, commanding the center of the painting.

During his time in Liberia, Epp created a colorful body of “visual poetry” featuring both architectural studies and urban landscapes and images from what he has called the “green chaos” of the West African jungle. In an interview in the *Vancouver Sun*, Epp recounted that when he worked in Liberia in the 1980s, he had heard about periodic small outbreaks of Ebola among humans in central Africa. The news of the 2014 Ebola outbreak in multiple West African countries motivated him to find a way to help a country and a region important to him as an artist and to his family. In November 2014, Epps worked with the Vancouver-based Marion Scott Gallery/Kardosh Projects on an exhibition and sale of nearly 40 his paintings from Liberia. All proceeds from the gallery’s sale of his works and a portion of the artist’s proceeds were subsequently donated to support the work of Medécins Sans Frontières in responding to the Ebola epidemic.

Since that time, the situation in West Africa has evolved. The World Health Organization (WHO) declared Sierra Leone free of Ebola virus transmission on November 7, 2015, and Guinea free of Ebola virus transmission on December 29, 2015. On January 14, 2016, WHO declared that human-to-human transmission of Ebola had also ended in Liberia—the country that has experienced the highest number of deaths from Ebola. Researchers now know that Ebola virus can persist in certain parts of the body in some persons who have recovered from the disease. Continued vigilance in West Africa remains a public health priority.

## Footnotes

1 Personal communication with Edward Epp, December 7, 2015.
